# Conversation Within a Facebook Smoking Cessation Intervention Trial For Young Adults (Tobacco Status Project): Qualitative Analysis

**DOI:** 10.2196/11138

**Published:** 2018-09-04

**Authors:** Karma McKelvey, Danielle Ramo

**Affiliations:** ^1^ Division of Adolescent Medicine Department of Pediatrics Stanford University Palo Alto, CA United States; ^2^ Center for Tobacco Control Research and Education University of California San Francisco San Francisco, CA United States; ^3^ Weill Institute for Neurosciences Department of Psychiatry University of California San Francisco San Francisco, CA United States

**Keywords:** Facebook, intervention, qualitative analysis, smoking cessation, social media, young adults

## Abstract

**Background:**

Smoking cessation interventions delivered through social media have the potential to engage young people in behavior change.

**Objective:**

The aim of this study was to describe participant-posted messages in a Facebook smoking cessation intervention for young adults to discern support for behavior change.

**Methods:**

We qualitatively analyzed data from the treatment arm of a randomized trial testing the efficacy of the Tobacco Status Project Facebook intervention. Young adults (N=138) aged 18-25 years (female: 81/138, 58.7%; white: 101/138, 73.2%; mean age 21 years) were recruited using Facebook and placed into one of the 15 secret Facebook groups based on readiness-to-quit smoking. Messages posted to groups for 90 consecutive days were tailored to readiness-to-quit: Not Ready (46/138, 33.3%), Thinking (66/138, 47.8%), and Getting Ready (26/138, 18.8%). Groups were randomized to receive up to US $90 for posting or no incentive. Two independent coders conducted open coding of user posts. We considered content by readiness-to-quit group and incentive condition.

**Results:**

There were 4 dominant themes across all groups: coping skills, friends and family, motivation to quit, and benefits of quitting. The dominant themes in Not Ready groups were friends and family (incentive) and motivation to quit (no incentive), whereas coping skills was the dominant theme in Thinking and Getting Ready groups. The expression of themes varied by readiness-to-quit group but not by incentive condition.

**Conclusions:**

Intervention messages tailored to readiness-to-quit appear useful in eliciting the desired responses from young adult smokers, with limited influence by monetary incentive.

**Trial Registration:**

ClinicalTrials.gov NCT02207036; https://clinicaltrials.gov/ct2/show/NCT02207036 (Archived by WebCite at http://www.webcitation.org/722XAEAAz)

## Introduction

Nearly all smokers (98%) begin smoking in adolescence and young adulthood (before the age of 26 years) [1]. Despite being just as motivated to quit as other adults and the wide availability of evidence-based smoking cessation interventions including quit lines, counseling, and medication, young adults are less likely to use these strategies to quit smoking than adults of other ages [1-4]. While emerging evidence shows that Web-based smoking cessation interventions have high user satisfaction and are effective for adults [5-7], among younger adults’ adherence to and engagement in online smoking cessation interventions remains low [8-11]. Still, with extremely wide use among young adults (88% of Americans aged 18-29 years in 2016) [12], Facebook may serve as an engaging tool, with broad reach, to deliver evidence-based smoking cessation interventions to this population.

Most research analyzing the content of digital interventions for smoking cessation has focused on quantitative analyses of data from online cessation communities (eg, volume or timing of posting). Across cross-sectional and longitudinal studies, participant engagement with digital interventions is associated with and predictive of smoking cessation [7,13-18]. Online smoking cessation communities have also been evaluated using social network analysis, a tool that helps describe the patterns of social relationships that form between groups and individuals [19]. Social network analysis revealed that the online smoking cessation community QuitNet has the characteristics necessary to sustain the support and promotion of cessation and that Facebook interactions were centralized, with a small number of users (“superusers”) leading the communications [20] and that demographic characteristics and posting behavior were similar across free public and private Web-assisted smoking cessation communities [20,21].

There is a smaller literature using qualitative methods to analyze the content of online and social media-based smoking cessation interventions. Computer-driven techniques (eg, latent Dirichlet allocation, correlated topic modeling) have been used to analyze social media data and offer advantages over human coding in analyzing large datasets [22,23]. One report identified concepts and themes in peer-to-peer messages on QuitNet to discern which themes were associated with abstinence. Investigators identified 12 themes comprising 43 concepts and found that abstinence was associated with interpersonal themes such as social support and cessation progress [24]. Another study described the content of “first-posts” by members of StopSmokingCenter.net to determine what content garnered a response-post and found that *problems with quit attempts* received a response the most often [25]. A study using framework analysis to characterize the content of posts to the Facebook page of Crush the Crave, an intervention aimed at young adults, found that the main purpose of participant posts was cessation support and identified 7 subthemes: management of cravings, promoting social support, denormalizing smoking, providing health information, encouraging cessation, exposing tobacco industry tactics, and group stimulation [26].

By facilitating change talk and conversations about change and abstinence, social media-based cessation interventions have the potential to support the change process; however, it is not yet understood if this is so. Nevertheless, the text-based nature of social media interventions offers a unique opportunity to characterize the representations of smoking and the change process across the stages of change. Our group developed the *Tobacco Status Project* (TSP) smoking cessation intervention delivered entirely through Facebook. Results from the randomized controlled trial (RCT) evaluating TSP showed significantly greater biochemically verified abstinence from smoking at treatment end in those who received the intervention (8.3%) than in those who received referral to the National Cancer Institute Smokefree.gov website: 3.2%, odds ratio 2.52 (95% CI 1.56-4.04), *P*<.001 [27]. There were no 12-month treatment effects for reported abstinence (*P*=.74), reduction in smoking by ≥50% (*P*=.53), likelihood of having made a quit attempt (*P*=.39), or stage of change over time (*P*=.97); retention was 71%. Participants in TSP engaged more and rated the intervention more favorably than those in the control condition.

Until now, there have been no qualitative reports on the content of participant posts in smoking cessation interventions embedded entirely within social media (ie, Facebook) and findings could be used to maximize the effectiveness of intervention messages within the context of social media smoking cessation interventions. In this study, we examined the overall content of participant-generated posts from the RCT testing the effectiveness of TSP and identified recurrent themes within the 3 readiness-to-quit groups and across the incentive condition. We have used specific quotes to illustrate the nature of frequent themes in each group.

## Methods

### Participants and Procedures

Data derived from the treatment arm of an RCT testing the efficacy of TSP is described elsewhere [27,28]. Young adults aged 18-25 years, residing in the United States, who had smoked ≥100 lifetime cigarettes, and who smoked ≥3 cigarettes per week were eligible and were recruited using Facebook ads [27]. Informed consent to participate was obtained online through the study website. Three multiple choice questions confirmed the understanding of study risks; identity was verified by email or social media; and then the online baseline assessment link was emailed [27,28]. Participants were randomized to either the treatment condition (TSP) or the control condition (referral to the National Cancer Institute Smokefree.gov website). Within the TSP condition, participants were assigned to a private Facebook group based on and tailored to their readiness-to-quit smoking at enrollment (precontemplation: “Not Ready”; contemplation: “Thinking”; preparation: “Getting Ready”); participants were assessed using the *Stages of Change Questionnaire* [28]. Groups began on a rolling basis starting when the first participant had been waiting no longer than 2 weeks; thus, group size varied. Groups were open for the duration of the trial (12 months), although content was only generated by the study team for the first 3 months.

**Table 1:** Study sample characteristics (N=138) from Tobacco Status Project, a smoking cessation intervention delivered entirely through Facebook.

Characteristic			Value
Male, n (%)			57 (41.3)
Age (years), mean (SD)			20.8 (1.9)
Hispanic, n (%)			8 (5.8)
**Race, n (%)**			
	White	101 (73.2)	
	More than one race	20 (14.5)	
	Black	5 (3.6)	
	Native American	1 (0.7)	
	Asian	1 (0.7)	
	Other	10 (7.2)	
**Region, n (%)**			
	West	38 (27.7)	
	South	52 (38.0)	
	Midwest	32 (23.4)	
	Northeast	15 (10.9)	
**Annual household income, n (%)**			
	≤$40,000	94 (68.1)	
	$41,000 - $80,000	28 (20.3)	
	$81,000 - $200,000	15 (10.8)	
	Currently in school (full time or part time)	42 (30.5)	
	Currently employed (full time or part time)	89 (53.5)	
**Smoking characteristics**			
	Are you a social smoker? (yes), n (%)	101 (73.2)	
	Daily smoker, n (%)	121 (87.7)	
	Fägerstrom Test For Nicotine Dependence, mean (SD)	2.9 (2.1)	
	Number of years smoked, mean (SD)	2.8 (0.6)	
	Cigarettes smoked per day, mean (SD)	10.4 (6.3)	
**Readiness-to-quit group, n (%)**			
	Not ready to quit	46 (33.3)	
	Thinking about quitting	66 (47.8)	
	Getting ready to quit	26 (18.8)	
	Past month marijuana use (yes)	59 (42.8)	
	Past month hookah use	33 (23.9)	
	Past month vape or e-cigarette or e-hookah use	69 (50.0)	

At the group level, participants were randomized to receive a monetary incentive (daily, weekly, or monthly) for commenting on intervention Facebook posts (up to $90) or no incentive. The participant pool for this study included all participants assigned to receive no incentive and those assigned to receive a monthly incentive (N=138), and the majority were white (102/138, 74.0%) and female (84/138, 60.0%; see Table 1 for detailed demographics and smoking characteristics).

Participants received 90 consecutive daily intervention posts (see Multimedia Appendix 1 for sample intervention posts) and “live” weekly one-hour counseling sessions during which a counselor, using Facebook commenting features, could answer participant questions in real time; and for those in the preparation stage of change at baseline, 7 state-of-the-art group cognitive-behavioral sessions were delivered through private Facebook groups. Intervention posts, including textual content, were designed and agreed upon before the study launch; dispatch of these posts was automated throughout the intervention according to the schedule. Trained intervention staff monitored the groups daily for any inappropriate content in responses to intervention posts. Additionally, doctoral-level trained smoking cessation counselors facilitated live weekly counseling sessions**.** Participants remained in the same group throughout the 3-month intervention. Daily posts employed the aspects of motivational interviewing, cognitive behavioral therapy coping skills, and the transtheoretical model (TTM) [29-31]. Posts to Not Ready groups were designed to enhance motivation and the importance of quitting, as well as to identify problems related to smoking. Posts to Thinking groups emphasized challenging the cons to change, the benefits of quitting, and TTM processes of change including consciousness-raising (learning new facts, ideas, and tips that support the behavior change), and making a small commitment to change. Posts to Getting Ready groups provided strategies for long-term smoking cessation together with making a commitment to quit, including setting a quit date and making a detailed plan for quitting, removing smoking paraphernalia from the home, and engaging in alternative behaviors. All posts included an image with text designed to elicit a response from participants (Multimedia Appendix 1).

### Analysis

First, data were downloaded from the Facebook app program interface, which was accomplished using “tools” from Facebook’s API Explorer. Data extraction included use of Facebook “access tokens,” which allowed study personnel to extract textual data from selected fields (all fields containing textual data were selected) within each group. Next, these data were provided to coders in a spreadsheet form with columns representing chosen fields. Next, two coders independently identified themes in transcripts using inductive or “open” coding. Open coding is data driven, that is, codes are based in the data (a “bottom-up” approach) rather than a theory-driven (“top-down”) one [32]. Investigators met after each transcript was coded to compare themes, resolve any discrepancies, and refine codes or themes. When it was agreed that thematic saturation had been reached (no emergent codes), a codebook containing identified codes was agreed upon.

Thematic analysis [33] was conducted first among all data then by readiness-to-quit group and incentive condition. Thematic “prevalence” (defined as the number of posts containing a particular theme) was calculated to account for differences in group size (number of participants) to identify and interpret dominant themes. Themes were described and patterns of themes within the data were examined and summarized to interpret their broader meanings and associated implications. All analyses were performed using Dedoose version 7.5.14 (SocioCultural Research Consultants, Los Angeles, CA, US) or Excel 2013 (Microsoft Corporation, Redmond, WA, US) [34].

## Results

### Findings

**Figure 1:**
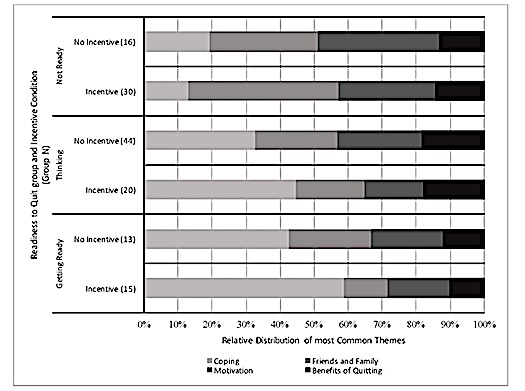
Dominant themes by readiness-to-quit group and incentive conditions from the Tobacco Status Project.

The 35 themes common to all groups are represented in Textbox 1. Four themes were most prevalent (“dominant themes”) across all readiness-to-quit groups and incentive conditions: (1) coping; (2) friends and family; (3) motivation; and (4) benefits of quitting. Within each readiness-to-quit group, differences in the frequency of themes varied negligibly by the incentive condition (Figure 1). The dominant themes in the Not Ready groups were friends and family in the incentive condition and motivation in the no incentive condition. The dominant theme for Thinking and Getting Ready groups was coping across incentive conditions. An examination of theme content across incentive conditions in all groups showed little variation by incentive, and thus, expression of codes was interpreted by theme and readiness-to-quit, rather than by incentive.

In response to any intervention content, 2517 posts (µ=503) were made in the Not Ready groups, 1687 in the Thinking groups (µ=281), and 1943 in the Getting Ready groups (µ=486), totaling 6147 comments with a mean of 423 comments per group. The average number of comments did not differ between the Not Ready and Getting Ready groups (independent sample *t*_7_=0.15, *P*=.89), Not Ready and Thinking groups (*t*_9_=1.56, *P*=.15), and Getting Ready and Thinking groups (*t*_8_=1.37, *P*=.21). Likewise, the average number of comments did not differ by incentive condition within Not Ready groups (*t*_3_=1.06, *P*=.37), Thinking groups (*t*_4_=0.09, *P*=.93), and Getting Ready groups (*t*_2_=0.88, *P*=.47).

### Coping

#### Not Ready

Posts coded as coping were ambivalent:

I enjoy walking, and watching movies or episodes of my favorite TV shows. But unfortunately as much as I enjoy those things, I don't see them holding my interest...

Male, 20 years

They were often pessimistic:

I can't think of something that would help me, except eating.

Female, 22 years

Posts containing outlandish suggestions were not uncommon:

Go climb a mountain!

Female, 22 years

Still, participants were able to name activities that could help them cope with smoking triggers and support quitting, as well as ways in which they currently cope with the drawbacks of smoking:

I like the extending time [between cigarettes] idea a lot...

Male, 20 years

I wear a separate shirt to smoke in...

Male, 20 years

At the same time, participants of the Not Ready groups directly negated the potential of coping strategies to be effective and the necessity of current strategies:

I've tried breathing exercises but none seem to be as instant as smoking...

Male, 20 years

Sometimes I spray perfume, but I usually don't care...

Female, 18 years

**The top 35 themes identified in the Tobacco Status Project.**

1. Alcohol
2. Big tobacco
3. Co-use
4. Cold turkey
5. Coping
6. Dependence
7. Dissonance
8. Drawbacks
9. Feedback
10. Flavors
11. Friends and family
12. History
13. Identification
14. Initiation
15. Media
16. Medication
17. Money
18. Motivation
19. Movies
20. Nicotine replacement therapy
21. Obstacles
22. Progress
23. Quit Benefits
24. Quit history
25. Secondhand smoke
26. Self-efficacy
27. Smoke-free policy
28. Smoker persona
29. Smoking benefits
30. Smoking legislation
31. Smoking norms
32. Social support
33. Teachable moments
34. Triggers
35. Vape

#### Thinking

Posts among participants in the Thinking groups mostly shared coping strategies that they had experience with and that worked for them:

Keeping hands busy, or keeping a tooth pick in my mouth helps. Also keeping something in my hand, like a cup of coffee or tea also helps...

Male, 22 years

Most the time when I was stressed I was just over thinking things so I just took a step bak [sic] n [sic] took a deep breath...

female, 23 years

Participant posts also revealed willingness to try strategies in the future:

I would choose to be more active and start jogging/running daily...

Male, 19 years

Posts generally had a supportive tone when making suggestions (on how to cope when quitting) to the group:

Get up and do something active and because it takes my mind to a different place then [sic] smoking...

Female, 24 years

Fill your free time with activities such as learning a new skill. Its [sic] hard to think about smoking when you’re immersed in concentration.

Male, 19 years

#### Getting Ready

Coping-coded posts for this group were rooted in the present insofar as participants posted predominantly about what they *are* doing:

Keeping them [cigarettes] all the way in my car really helps because I am really lazy lol...

Female, 22 years

I workout [sic] for half an hour and run two miles every day :)...

Male, 19 years

Posts were generally upbeat and often humorous:

Walk with two drinks in my hand. Can't hold a cigarette if your [sic] holding two drinks right?!

Male, 21 years

Go to my other addiction, the internet lol...

Male, 24 years

### Friends and Family

#### Not Ready

Friends and family coded posts indicated friends and family were seen as either supportive of participants’ quitting or indifferent to participants’ smoking:

My sister tells me she doesn’t want me to die...

Female, 19 years

They don't really say anything about it...

Female, 21 years

At the same time, participants indicated the potential of friends and family members as motivation to quit, and posts coded “friends and family” were more often than not coded also with “motivation”:

I can quit I just really dont want to unless my girlfriend gets pregnant...

Male, 20 years

#### Thinking

Posts coded as friends and family in the Thinking groups typically referred to friends and family members as people harmed by participants’ smoking or who may suffer as they quit smoking:

I think of my housemates and friends, whom I constantly smoke around. I always feel guilty when someone talks about secondhand smoke...

Male, 19 years

I'm worried about being mean to those I love.

Male, 19 years

A less-common yet consistent concern posted about was how to deal with the smokers in their lives:

I think it will be hard to hang out with a lot of my friends who smoke after I quit. A majority of my friends smoke so it might be difficult to get away from other people smoking...

Male, 21 years

Posts generally reflected ambivalence, consistent with “contemplating” quitting.

#### Getting Ready

For this group, most posts reflected the doubt participants perceived among their friends and family:

My support system was excited, skeptical but happy that I'll be quitting.

Female, 25 years

A few said I won't quit and it won't last.

Male, 21 years

They don’t believe me lol...

Female, 18 years

The remaining posts within this theme were mainly pragmatic—both when alluding to support and hindrance:

Luckily, im [sic] very close to family, so I will very likely go to my sisters [sic] or brothers [sic] for...support...

Male, 20 years

My girlfriend hasn’t helped much considering she asks me if I wanna [sic] smoke one when she gets home everyday [sic].

Male, 21 years

In addition to being pragmatic, posts reflected “when” participants would quit, not “if.”

### Motivation

#### Not Ready

Posts that coded motivation in the Not Ready groups were generally dual-coded with “friends and family.” Posts reiterated participants’ unwillingness to quit while citing friends and family or other things outside themselves that could be motivating in the future:

It’s not really important to me right now, but it’s a 10 [on a scale of 1 to 10] to quit before I have children...

Female, 21 years

Although some posts were solely about participants’ (lack of) motivation:

Well we all die in the end. Smokers and non smokers [sic]. Morbid, I know but. [sic] #sorryboutit...

Male, 22 years

#### Thinking

Most posts that coded motivation in the Thinking groups referred to future or external motivations to quit. Saving money and health were common motivations to quit smoking:

I’d only like to quit for the financial benefits really...

Male, 21 years

It's a horrible habit and I want to quit. For health reasons alone!

Female, 23 years

Secondarily, benefit to family (including pets) and friends was cited as motivation to quit:

My dogs honestly! Lol everyone in this house smokes except them, and they can't exactly crack the window now can they?

Female, 23 years

I think about my friends that don't smoke and my little cousins.

Female, 23 years

Posts often included ≥2 of these ideas:

More money, better health...

Male, 21 years

I would definitely have more money to buy other things that are needed like planning [sic] my little girls [sic] first birthday and helping my husband see the importance of quitting for her and our health reasons...

Female, 21 years

#### Getting Ready

Motivation-coded posts for Getting Ready groups were emphatic and predominantly self-focused:

I want to be able to run faster for longer. I want to be dependent on nothing. I want to make my Dad proud.

Male, 20 years

My lung capacity. Not coughing up stuff.

Male, 21 years

...then I coughed up blood. Nope.

Male, 19 years

I’ve been going to the gym 3 times a week!

Male, 24 years

Participants’ health was the unifying thread here:

My health. My health. My health.

Female, 22 years

### Benefits of Quitting

#### Not Ready

Most posts specified health or monetary benefits associated with quitting and were future-focused, yet some had a skeptical tone:

I could swim again. Without DYING...

Male, 22 years

Probably be healthier. Feel better throughout my day.

Male, 18 years

Many of these posts referenced participants’ image, particularly with regard to smelling like smoke:

I won’t smell “icky”...

Male, 20 years

Other than the occasional use of “would”, posts were devoid of language indicating participants thought for certain that their health (or sport or recreational activity performance, voice, sensory perceptions) would improve with quitting smoking:

My teeth would look better and I would have more money...

Female, 21 years

And chances are you'd be able to smell smoke a lot easier...

Female, 21 years

#### Thinking

Benefits of quitting posts revealed that participants in this group were looking most forward to being free from (nicotine) dependency or withdrawals, breathing easier, and having more money. All posts coded with benefits of quitting for the Thinking groups listed >1 benefit:

I would be able to breath [sic] better, do more activities because I can breath better and I would save a lot of money every year...

Female, 23 years

Best case senario [sic], better health, no taking time away from work or hanging out to go smoke, more money, less debt, freedom...

Female, 23 years

I would lose dependency on an item when I'm stressed or hurt and could actually face what was causing those feelings right away. And I would save so much money.

Female, 18 years

Generally, these posts were optimistic and enthusiastic:

Everything will be better! My teeth, my breathing, my health in general, my wallet, and just feeling great about myself knowing that nicotine doesn't control me anymore!!!

Female, 19 years

#### Getting Ready

Benefits-coded posts in the Getting Ready groups had a sense of impending liberation and referred to improved health and demeanor:

I will definitely feel my stamina come back when i [sic] quit. I love to run and play soccer.

Male, 20 years

No smoke smell on my clothes.

Female, 22 years

Saving money and my health having better self-discipline and not giving into an unhealthy coping mechanism [sic] ...Working out and not dying...

Female, 20 years

Participants had a tendency to incorporate comparisons of their current self-image with images after quitting:

Freedom, I won't smell, more energy, won't be SOB...

Female, 25 years

...being able to breathe better! i [sic] get winded so easily...

Female, 22 years

## Discussion

### Principal Findings

To begin to understand how social media-based interventions may support behavior change (ie, quitting smoking), we examined the content and volume of participant posts from TSP, a smoking cessation intervention for young adults delivered entirely through Facebook. We identified dominant themes throughout by readiness-to-quit group and by monetary incentive for engagement. There were slight variations in the content expressed in groups across readiness-to-quit and incentive conditions, and young adult smokers were most likely to post content related to motivation to quit, coping strategies, exploring relationships with friends and family members in the context of quitting, and exploring the benefits of cessation in all groups. These topics are consistent with the overall intention of the TSP intervention to enhance motivation, support change talk, and promote the use of coping strategies for cessation.

While all 3 readiness-to-quit groups across incentive conditions had the same 4 dominant themes, how these themes manifested in each group was quite different. Consistent with stage-matched intervention theory and the tailored content within intervention posts, participant posts in the Not Ready groups were related to raising doubt about continuing to smoke and enhancing motivation through values evaluation. In more motivated groups, participant posts were more focused on strategies for coping with quitting (eg, changing behaviors to ameliorate effects of withdrawal) and less on motivation. Findings show that participant behavior in the context of private social media groups is consistent with the intent of in-person smoking cessation interventions tailored to readiness-to-quit smoking and support online delivery of such tailored interventions.

Across incentive conditions and themes, those in Not Ready groups reiterated their unwillingness to quit smoking now. Posts in the Not Ready groups consistently contained qualifier words such as “if,” “but,” “might,” “maybe,” and “sometimes” when responding to intervention posts suggesting possible ways to change their smoking behavior. This is consistent with a hesitant stance favoring no change and speaks about the importance of using motivational interviewing techniques such as expressing empathy and rolling with resistance, even in the context of social media posts [35]. The posts with content coded “family and friends” also typically contained content coded “motivation” in the Not Ready groups, which contextually suggested that focusing on family and friends may function as a barrier to change for participants not ready to quit. Content emphasizing the availability of social support through a social media smoking cessation intervention may be particularly effective for this group [36].

While participants in the Thinking groups shared their experiences of past quit attempts and indicated in their posts that they would be willing to try new behaviors, posts for this group had an ambivalent tone overall. Indeed, it is this ambivalence that can keep individuals stuck in this stage for long periods of time [37]. Furthermore, just as these participants posted about how they felt smoking had affected the people in their lives, they tended to focus on how quitting could benefit these people (versus themselves). This external focus could also be an indication that smokers in contemplation have yet to internalize the benefits of quitting for themselves [38]. Still, many Thinking posts referred to participants’ wish to be free from nicotine dependence; targeting this group with messaging focused on freedom from addiction could aid in moving them toward action (quitting).

Posts in the Getting Ready groups were emphatic and permeated by levity. For example, posts evidenced doubt among participants’ social groups that they would be successful in quitting. These misgivings were addressed pragmatically and with humor by participants and seemed to have motivated rather than discouraged participants in their efforts to quit. Furthermore, posts indicated that participants were internally focused: they described actions they were already taking and posted about how better they felt and how they would feel upon successfully quitting, indicating perceived self-efficacy, which is a necessary component of successful behavior change [37-39]. The usefulness of tailored TSP posts to this group is exemplified by participants’ drive to stay the course of abstinence to achieve freedom from dependence. Employing the same or similar messaging in future social media interventions for young adults is warranted.

There were only negligible differences in dominant themes between incentive conditions, suggesting that the content was not altered by the presence of a monetary incentive tied to engagement. This finding supports the literature suggesting that incentives do not undermine participants’ intrinsic motivation to change health behaviors; unlike other behaviors, which have shown an undermining effect of incentives [40]. In the TSP-evaluating RCT, incentives were found to be related to comment volume in contemplation (χ^2^=14.59, df=2, *P*=.002) and preparation (χ^2^=9.95, df=2, *P*=.02) but not precontemplation (χ^2^=6.80, df=2, *P*=.08), suggesting that there is some promise for using monetary incentive to increase the number of participant posts in social media-based smoking cessation trials without an associated impact on quality [27]. Limited differences in the expression of common themes in Not Ready and Thinking groups suggest that content could potentially be merged in future interventions.

### Limitations

The themes identified in participant comments were guided in part by the content of the posts themselves. However, given the nascent literature on behavior in social media intervention, it was not clear whether the content would be germane to the intervention. Participant posts from The Doctor Is In sessions were not investigated independently of daily intervention posts. The results of our analysis of data from a Facebook intervention for young adult smokers may not generalize to other social media platforms (eg, Instagram), user profiles (eg, older adults), or health risk behaviors.

### Conclusions

Overall, tailored messaging delivered through a social media smoking cessation intervention appears to support content reflective of the theories driving the intervention across all stages. As social media continues to be a resource for engaging young adults in healthy behavior change, qualitative analyses can inform treatment targets and show that tailoring interventions to readiness to change is likely an ideal strategy for enhancing motivation and supporting behavior change.

## Appendix

Multimedia Appendix 1 Sample intervention and participant posts from each of the four most common themes across readiness-to-quit groups in the Tobacco Status Project.

## Abbreviations

TSP: Tobacco Status Project
TTM: transtheoretical Model
RCT: randomized controlled trial
