# Supplementary material for: Conversation Within a Facebook Smoking Cessation Intervention Trial For Young Adults (Tobacco Status Project): Qualitative Analysis
Source: JMIR Form Res. 2018 Sep 4;2(2):e11138. doi: 10.2196/11138 (PMC6334697; doi:10.2196/11138)
Supplement: Multimedia Appendix 1 [file formative_v2i2e11138_app1.pdf]

	Not Ready	Thinking	Getting Ready
**Coping**	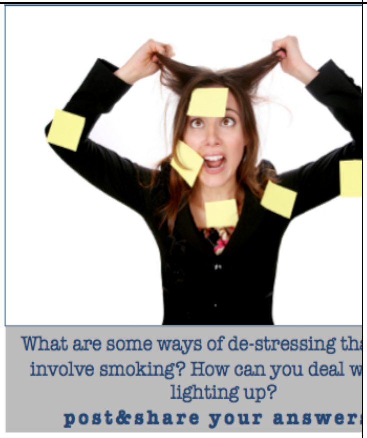<br><br><br>Most people who smoke report dealing with stress as their top reason for smoking. Work, class, family, friends, significant others -- it can be a lot! Can you think of another way to deal with stress that doesn't involve lighting up when things get tough? What could you do instead?<br><br>*Slapping people or going for a drive* (male, 20)	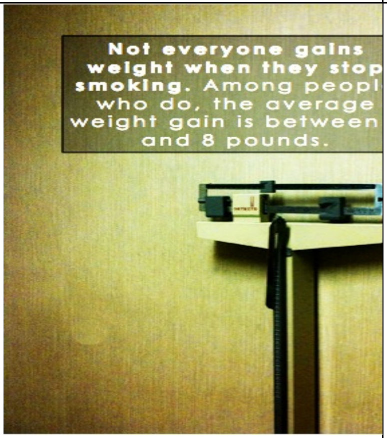<br><br><br>Weight gain is one of the top concerns for almost every smoker. We've made a list of some tips that can help keep your weight on track while you quit. Pick the one sounds good to you. How could you implement it into your life?<br><br>*Well I already work out multiple times a day and drink lots of water, my plan when I do quit is to just turn the excess fat into muscle* (male, 21)<br><br>*I just eat a whole lot of candy and coffee to get me through the first few weeks and then start hitting the gym as an alternative and loose the 5 pounds I gained.* (female, 21)	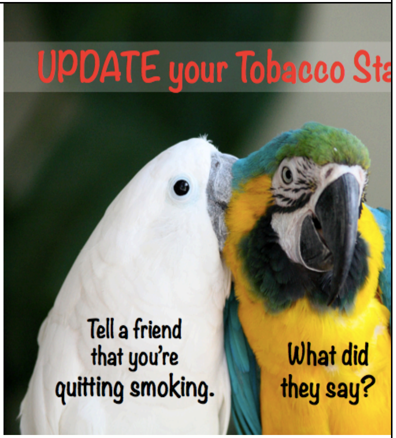<br><br><br>Post what happened when you told your one (or a few) friends about your plan to quit.<br><br>*Well I told my dad, shook his hand, and he said he wasn't giving me a single cigarette again.* (male, 19)

	Not Ready	Thinking	Getting Ready
**Friends and Family**	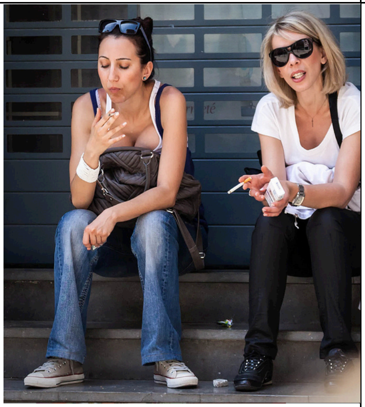<br>Why might your smoking buddies want you to keep smoking? What are their motives?<br><br>*If I wanted to quit they wouldn't be dicks. They are my friends and they love and support me. If your smoking buddies don't support you... They aren't your friends and forget them for real though.* (male, 20)	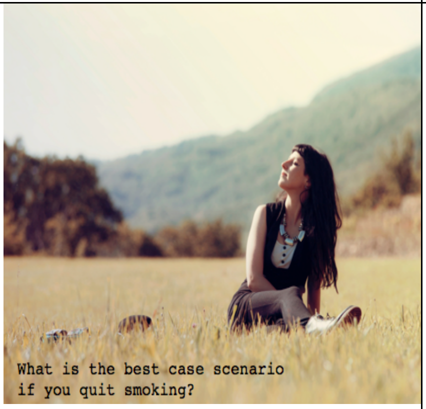<br><br>What would be the best-case scenario if you quit smoking?<br><br>*My daughter won't have to worry about having problems breathing because of her mother. Or being made fun of for smelling like smoke like i was in school. I want to be healthy and be able to care for her in my best health!* (female, 18)<br><br>*My struggle is that every one of my friends smoke so when I'm alone I don't have the craving. I don't smoke. But when I'm with friends who constantly chain smoke and I smell it, I love the smell of the cigarettes I smoke.* (female, 19)	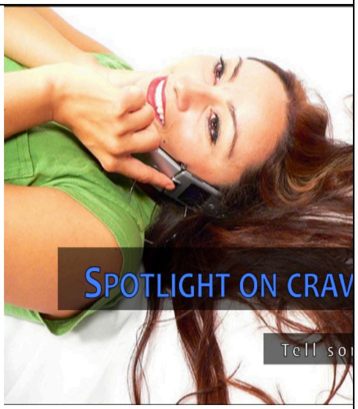<br><br>When your cravings are getting bad, call someone you trust. Tell them how you're feeling and that you don't want to smoke. Try it out and let us know how it went.<br><br>*My boyfriend is quitting with me now!!! Extra motivation, Calling him helps a lot <3* (female, 20)

	Not Ready	Thinking	Getting Ready
**Motivation**	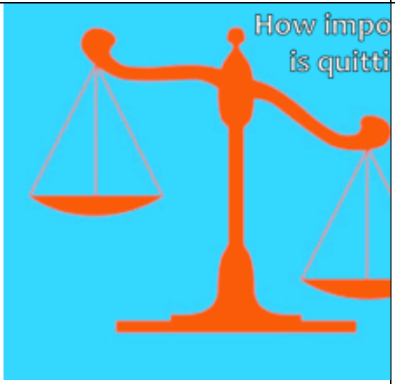<br>On a scale of 1-10, how important is it to you to quit smoking for good?<br><br>*Yeah, i quit whenever I was pregnant. But I started again when she was born mainly because of ppd* [post partum depression]*. When I have another child, I might just quit for good.* (female, 22)	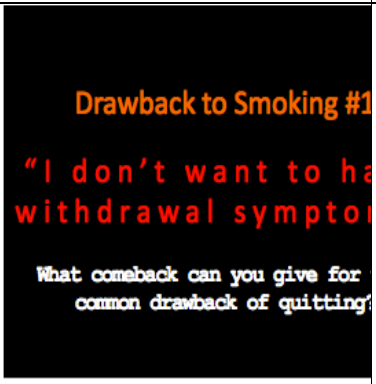<br>Research shows that emphasizing the benefits of quitting over the drawbacks helps smokers quit their habit for good. Try your hand at making a negative a positive. What's your comeback for this drawback?<br><br>*Seeing friends quit heroine* [*sic*] *make quitting cigarettes look like a cake walk. I think I can handle being agitated for a little while* (male, 22)	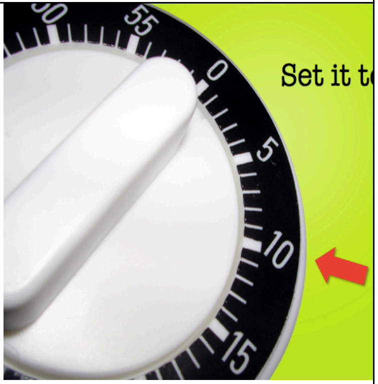<br>Practice makes perfect! Today, everytime you feel the urge to light up, wait at least 10 minutes and practice using a smoking substition. What did you choose to do, and how helpful was it to curb your craving?<br><br>*I'm never smoking again. Ef tobacco, its useless, pointless. I can literally feel the damage its doing to my body. Its gives me panic attacks. I don't need this. My body shakes when I smoke. I hate it.* (male, 19)

<table>
<tr><th></th><th>Not Ready</th><th>Thinking</th><th>Getting Ready</th></tr>
<tr>
<td>Benefits of Quitting</td>
<td>
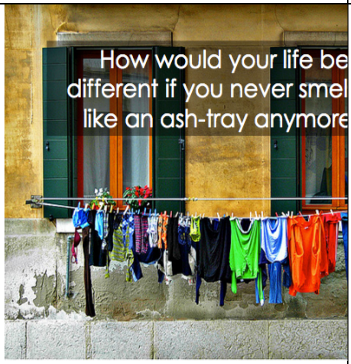

How do you think your life would change if you always smelled fresh and never like a cigarette?
I wouldn't be so worried about how I smell at work and I wouldnt have to constantly change my shirt to smoke and wash my hands after to hold my son. Oh and I wouldn't have to deal with my fiancee saying how much she hates the smell lol (male, 23)
My friends' parents wouldn't think I'm some street ruffian. (male, 18)
</td>
<td>
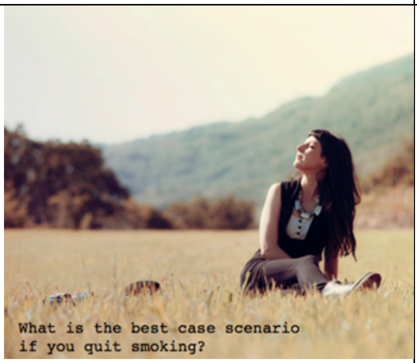

What would be the best-case scenario if you quit smoking?
Saving my hard earned money to put toward other things. Along with a better quality of life plus better health overall. (female, 19)
Best case senario [sic], better health, no taking time away from work or hanging out to go smoke, more money, less debt, freedom (female, 23)
</td>
<td>
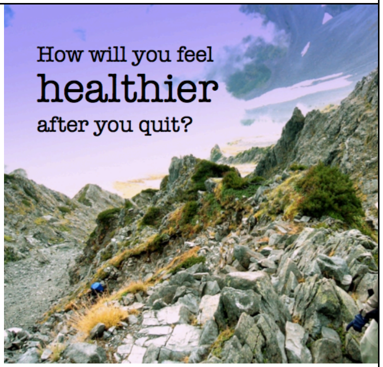

The following post was made by a successful quitter: "I focused every day on another way that I felt better. One day, I'd notice how much easier it was to breathe, and another day I would realize how I could walk up the stairs without getting winded. Reminding myself each day about what I was getting out of quitting made it seem worthwhile." How do you think you'll notice your body getting healthier when you quit?
I think it will most benefit my mental health when I am finding better coping mechanisms for my anxiety. (female, 22)
Not hacking my lungs up after running. (female, 20)
</td>
</tr>
</table>
